# Supplementary figures and images for: A high-throughput platform for single-molecule tracking identifies drug interaction and cellular mechanisms
Source: eLife. 2025 Jan 9;12:RP93183. doi: 10.7554/eLife.93183 (PMC11717362; doi:10.7554/eLife.93183)

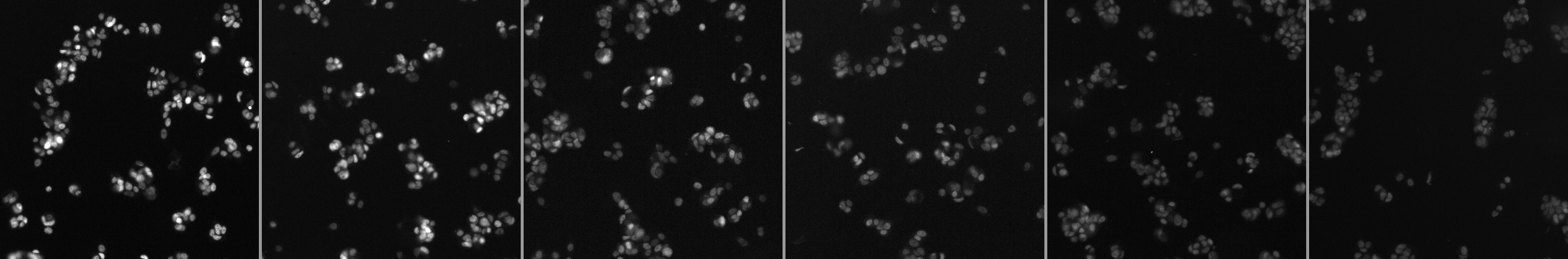

Supplement: Figure 5—source data 1. [file elife-93183-fig5-data1.zip › Figure5_supFigure1_sourceImgs/MCF7_montage_dm.tif]

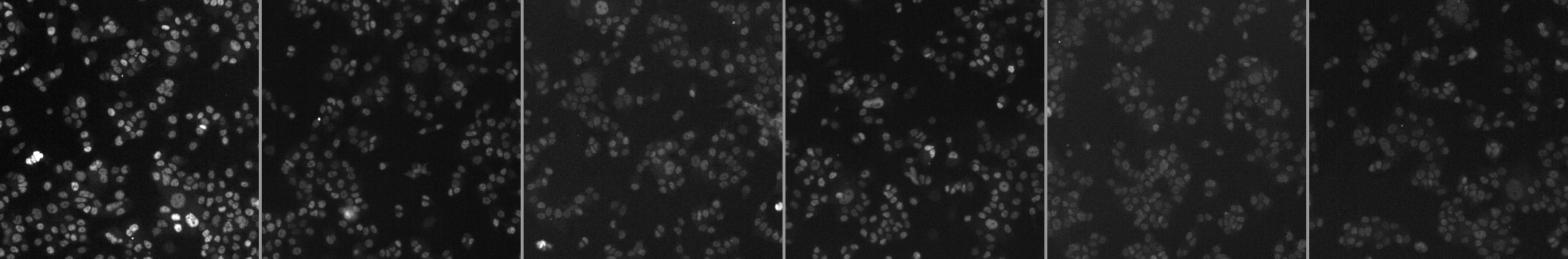

Supplement: Figure 5—source data 1. [file elife-93183-fig5-data1.zip › Figure5_supFigure1_sourceImgs/T47d_montage_dm.tif]

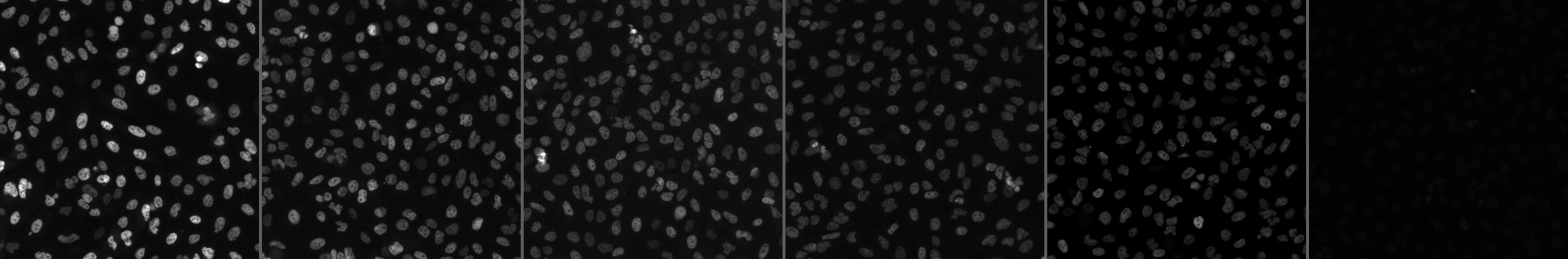

Supplement: Figure 5—source data 1. [file elife-93183-fig5-data1.zip › Figure5_supFigure1_sourceImgs/U2OS_montage_dm.tif]

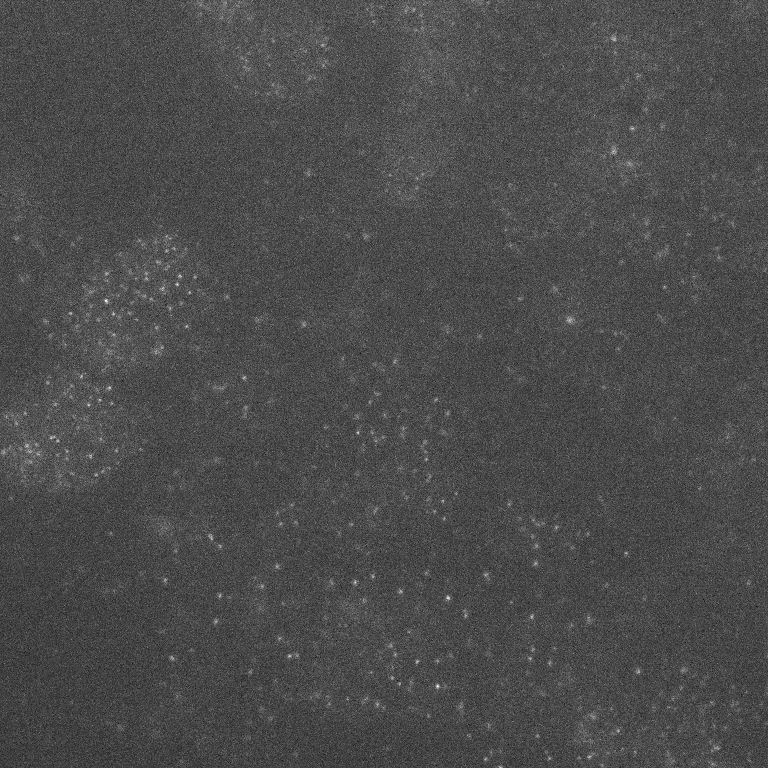

Supplement: Figure 5—source data 3. [file elife-93183-fig5-data3.zip › Fiigure1 - sourceImgs/716634-U2OS-0010_WellB19_Point01_ChSPT_overlay.tif]

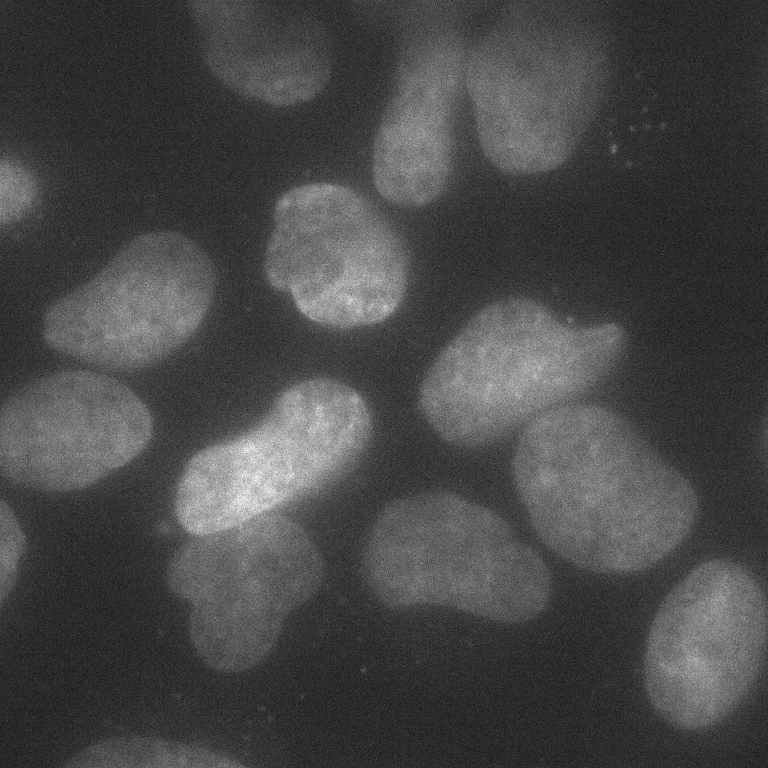

Supplement: Figure 5—source data 3. [file elife-93183-fig5-data3.zip › Fiigure1 - sourceImgs/AVG_716634-U2OS-0010_WellB19_Point01_ChMask1.tif]

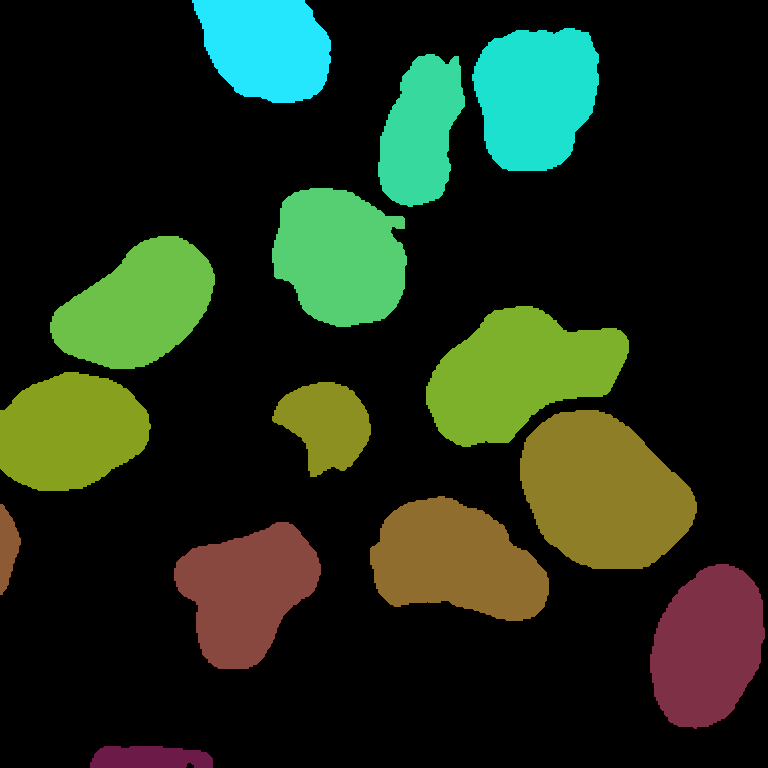

Supplement: Figure 5—source data 3. [file elife-93183-fig5-data3.zip › Fiigure1 - sourceImgs/Mask_B19_1.png]

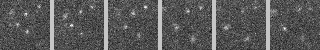

Supplement: Figure 5—source data 3. [file elife-93183-fig5-data3.zip › Fiigure1 - sourceImgs/Montage_CaaX_2.tif]

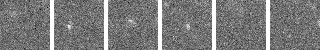

Supplement: Figure 5—source data 3. [file elife-93183-fig5-data3.zip › Fiigure1 - sourceImgs/Montage_FreeHalo.tif]

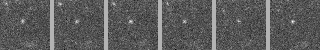

Supplement: Figure 5—source data 3. [file elife-93183-fig5-data3.zip › Fiigure1 - sourceImgs/Montage_H2B.tif]

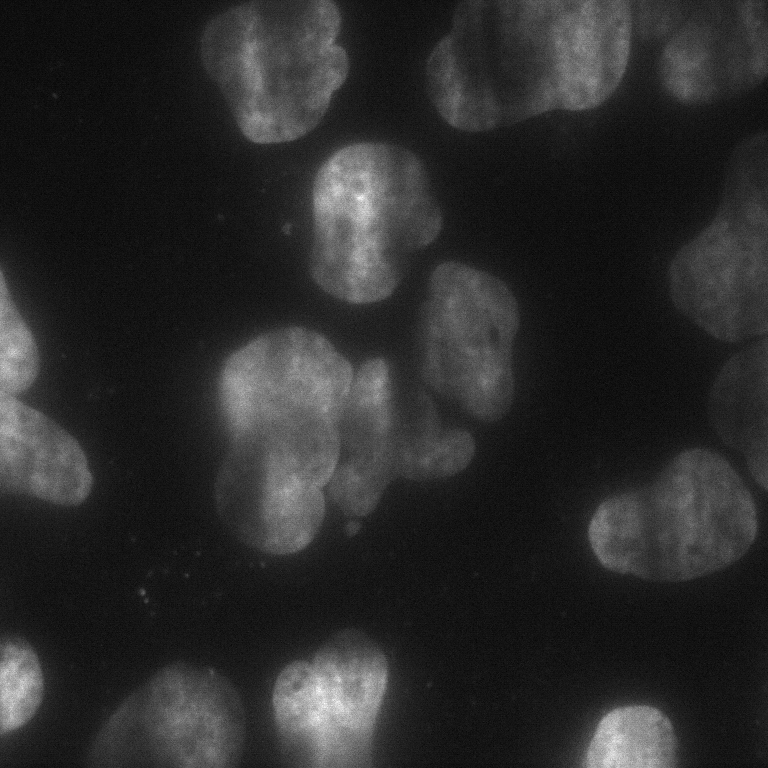

Supplement: Figure 5—source data 3. [file elife-93183-fig5-data3.zip › Fiigure1 - sourceImgs/740719-U2OS-0016_WellH13_Point05_ChMask1_corr.tif]

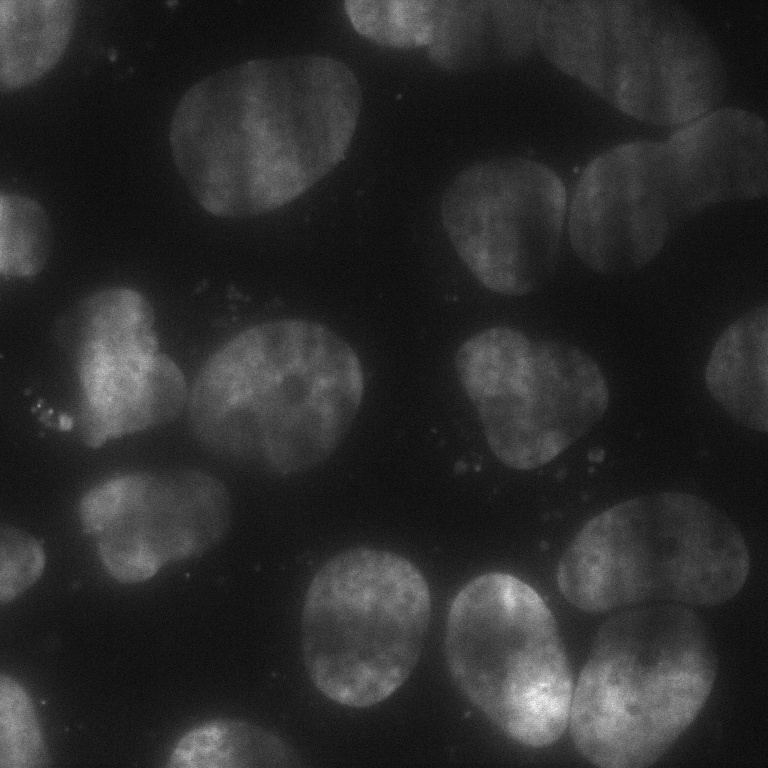

Supplement: Figure 5—source data 3. [file elife-93183-fig5-data3.zip › Fiigure1 - sourceImgs/740719-U2OS-0016_WellF10_Point03_ChMask1_corr.tif]

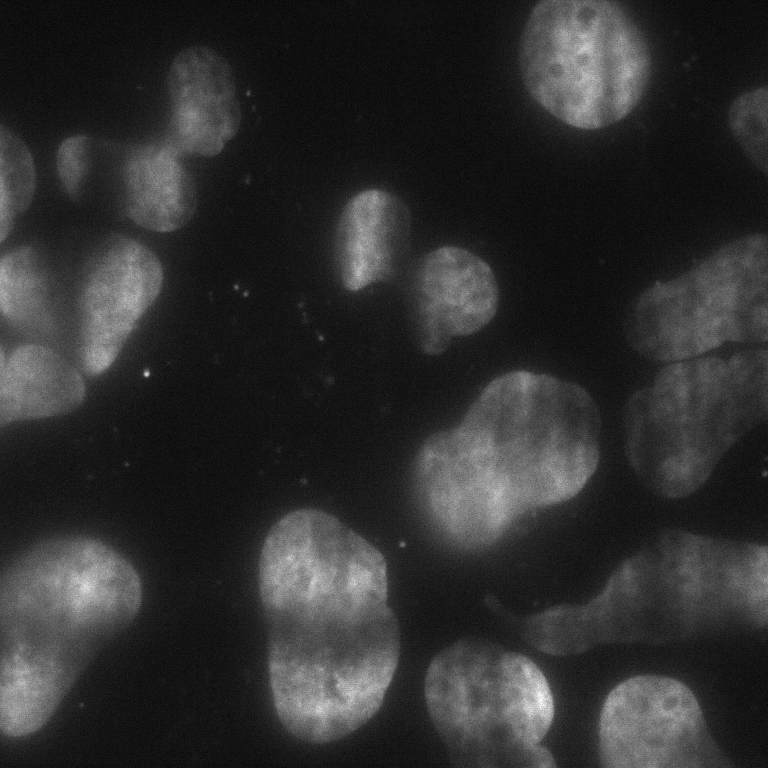

Supplement: Figure 5—source data 3. [file elife-93183-fig5-data3.zip › Fiigure1 - sourceImgs/740719-U2OS-0016_WellB12_Point05_ChMask1_corr.tif]

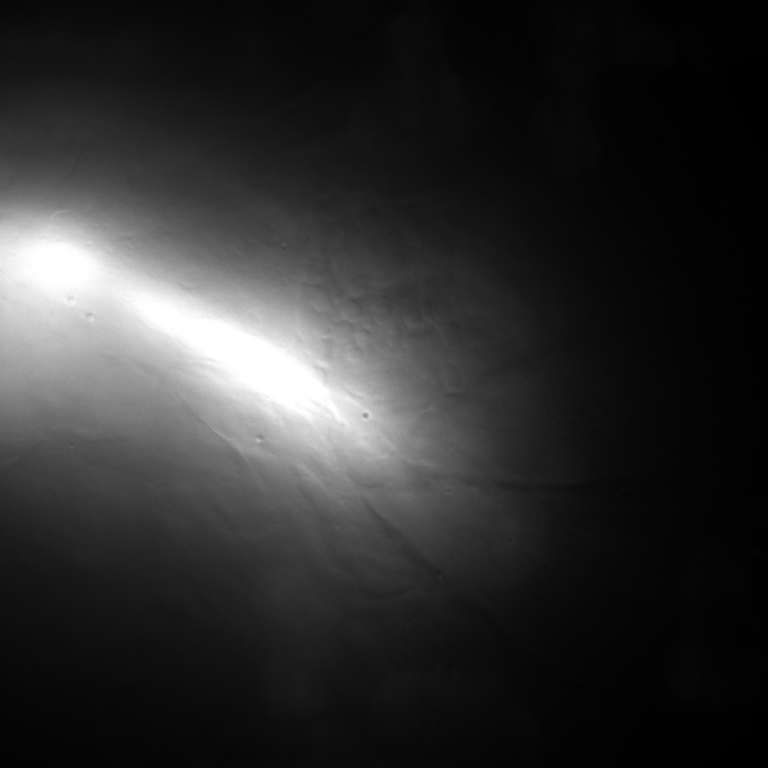

Supplement: Figure 5—source data 3. [file elife-93183-fig5-data3.zip › Fiigure1 - sourceImgs/740719-U2OS-0016_WellB12_Point01_ChMask1_corr.tif]

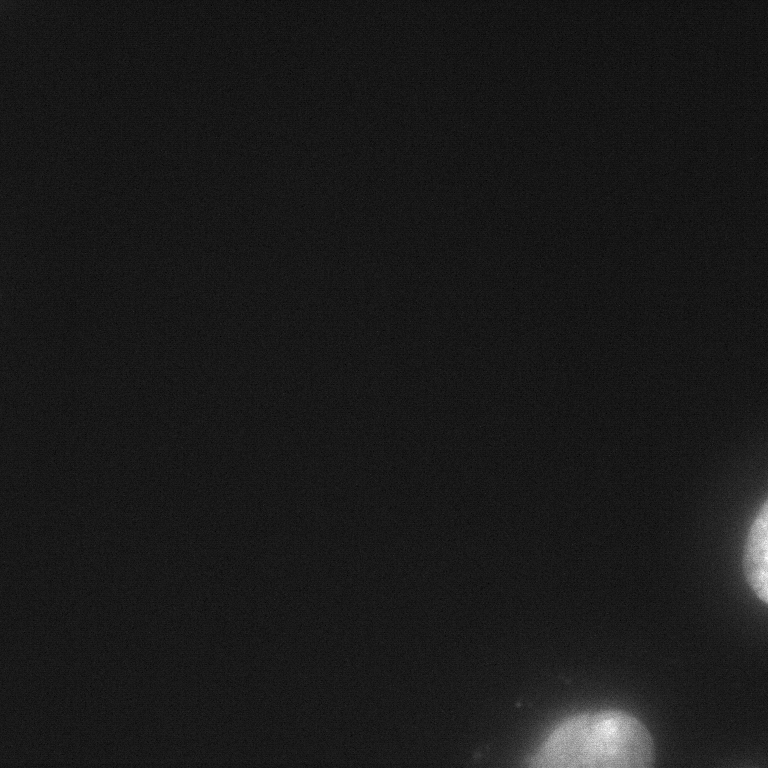

Supplement: Figure 5—source data 3. [file elife-93183-fig5-data3.zip › Fiigure1 - sourceImgs/740719-U2OS-0016_WellB08_Point01_ChMask1_corr.tif]

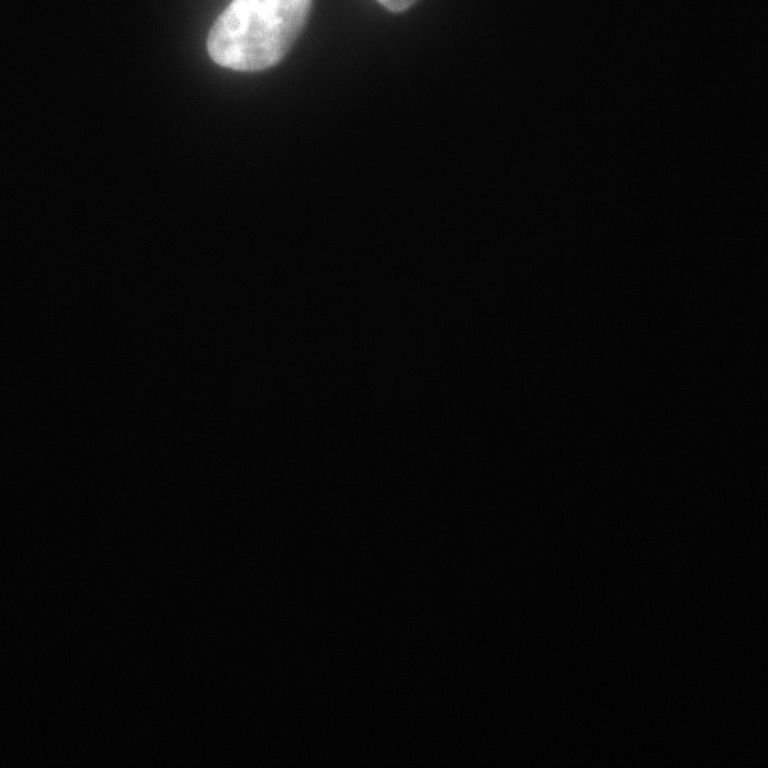

Supplement: Figure 5—source data 3. [file elife-93183-fig5-data3.zip › Fiigure1 - sourceImgs/616449-U2OS-0005_WellB04_Point00_ChMask1_corr.tif]

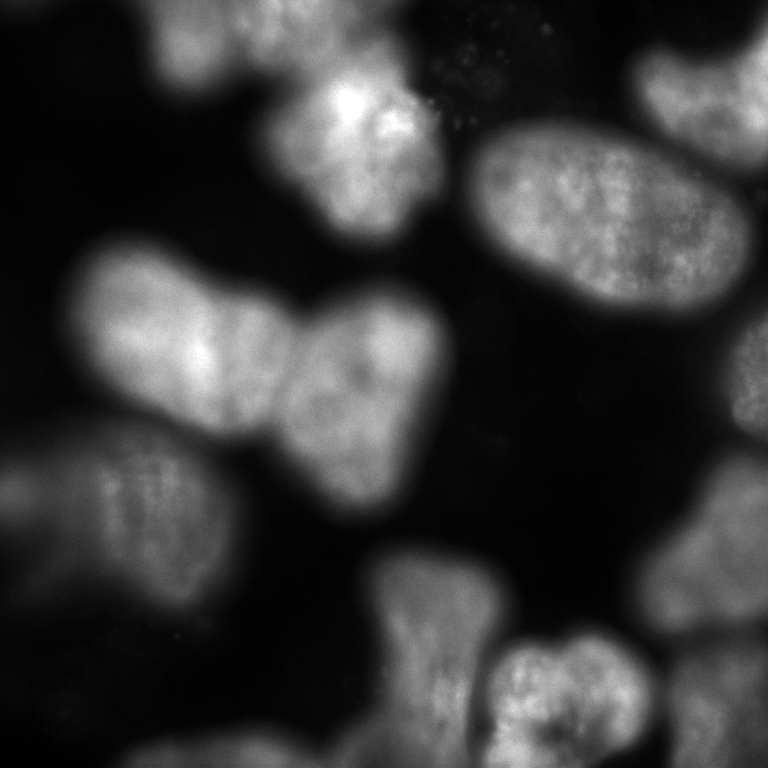

Supplement: Figure 5—source data 3. [file elife-93183-fig5-data3.zip › Fiigure1 - sourceImgs/616449-U2OS-0001_WellB03_Point02_ChMask1_corr.tif]

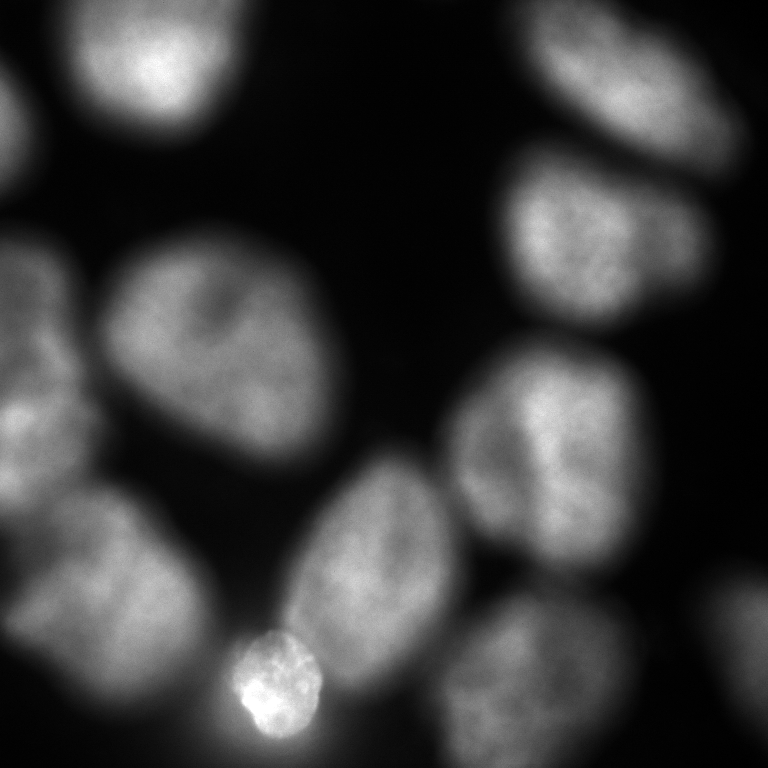

Supplement: Figure 5—source data 3. [file elife-93183-fig5-data3.zip › Fiigure1 - sourceImgs/616449-U2OS-0001_WellB02_Point01_ChMask1_corr.tif]

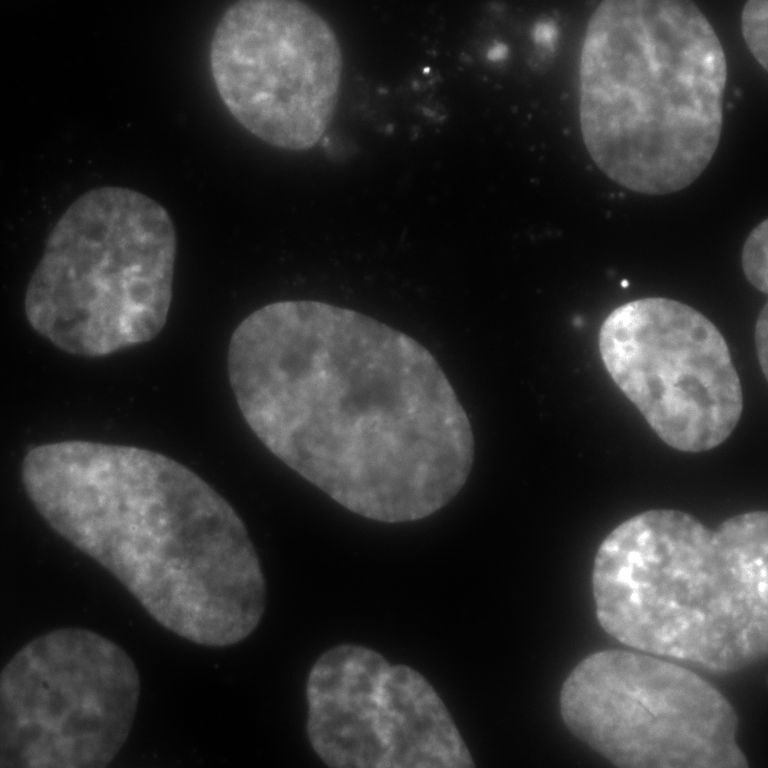

Supplement: Figure 5—source data 3. [file elife-93183-fig5-data3.zip › Fiigure1 - sourceImgs/567353-ERL10-0006_WellE07_Point02_ChMask1_corr.tif]

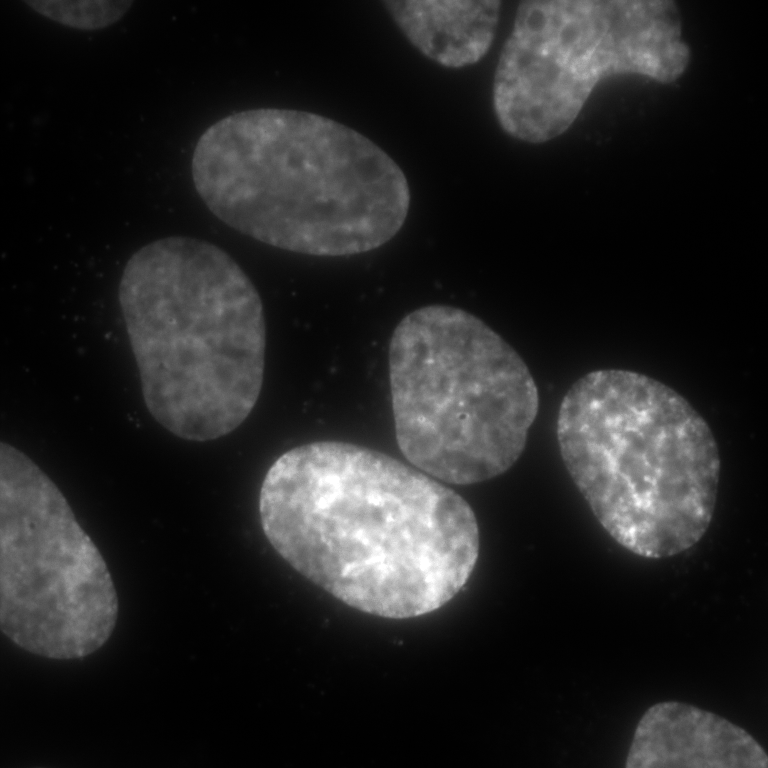

Supplement: Figure 5—source data 3. [file elife-93183-fig5-data3.zip › Fiigure1 - sourceImgs/567353-ERL10-0006_WellB06_Point05_ChMask1_corr.tif]
